# Emergency renal autotransplantation for kidney salvage following iatrogenic renal artery occlusion after aortic stent deployment: A case report

**DOI:** 10.1016/j.eucr.2026.103509

**Published:** 2026-06-08

**Authors:** Hung Ngoc Pham, Anh Van Quoc Nguyen, Can Van Truong, Tuan Kim Nguyen, Viet Huu Quoc Phan, Tuan Minh Truong, Phuc Dai Hong Vo, Hieu Van Le, Xuan Thi My Hoang, Dung Duc Nguyen, Kha Cong Le Bui

**Affiliations:** a Department of Urology, Hue Central Hospital, Hue, 49135, Viet Nam; b Department of Thoracic - Cardiovascular Surgery, Hue Central Hospital, Hue, 49135, Viet Nam

**Keywords:** Takayasu arteritis, Renal autotransplantation, Renal artery occlusion, Endovascular complication

## Abstract

Renal autotransplantation (RAT) is an established reconstructive technique but is rarely used as an emergency salvage procedure. We report a 20-year-old male with Takayasu arteritis who developed acute left renal artery occlusion following endovascular stenting of infrarenal aortic stenosis. Imaging confirmed complete occlusion of the renal artery ostium due to stent coverage, precluding endovascular revascularization. Emergency nephrectomy and RAT to the right iliac fossa was performed. Postoperatively, renal function progressively recovered with stable graft perfusion on follow-up. This case highlights that RAT can be an effective salvage strategy in catastrophic renovascular complications when conventional endovascular rescue is not feasible.

## Highlights

• Iatrogenic renal artery occlusion after aortic stent in Takayasu arteritis.
• Endovascular recanalization was not technically feasible.
• Emergency renal autotransplantation enabled kidney salvage.
• Successful outcome achieved within a limited ischemic window.
• RAT may be considered in catastrophic renovascular complications.

## 1 Introduction

Takayasu arteritis (TAK) is a chronic granulomatous vasculitis of unknown etiology, affecting the biggest scaffolding arteries like the aorta and its major tributaries as well ^1^ Endovascular intervention has emerged as an attractive treatment option for symptomatic arterial stenosis in TAK, offering less invasive revascularization and rapid hemodynamic improvement in selected patients ^2^ However, endovascular procedures also carry inherent technical risks, particularly in patients with complex vascular anatomy ^3^^,^^4^ Among these, inadvertent coverage of branch vessels during stent deployment is a rare but serious complication. In such situations, endovascular revascularization may not be feasible, and alternative strategies are required.

Renal autotransplantation (RAT) is an established technique in urology for complex renovascular and ureteral reconstruction. However, its role as an emergency salvage procedure in acute vascular complications remains limited. We report a rare case who developed an accidental occlusion of the left renal artery orifice following abdominal aortic stenting in a young patient with TAK. The patient was successfully managed with emergency RAT.

## 2 Case presentation

A 20-year-old male with a known history of Takayasu arteritis was diagnosed with bilateral renal artery stenosis at the age of 15 and underwent bilateral renal artery stenting, with stable renal function maintained over five years. Three months prior to admission, follow-up imaging demonstrated progressive 65% stenosis of the infrarenal abdominal aorta. The patient had no significant comorbidities, no history of smoking or alcohol use, and no relevant family history. On admission, he reported fatigue and reduced exercise tolerance. Physical examination revealed diminished lower limb pulses and a significant blood pressure discrepancy between the upper and lower extremities. Laboratory findings were within normal limits, including a serum creatinine level of 88.8 μmol/L and C-reactive protein of 1.7 mg/L. Doppler ultrasound demonstrated a narrowed abdominal aorta with a peak systolic velocity of 348 cm/s. Computed tomography angiography confirmed 65% stenosis of the infrarenal aorta with irregular wall thickening suggestive of active vasculitis (Fig. 1A and B). Based on these findings, endovascular treatment was indicated. A balloon-expandable covered stent (BeGraft, Bentley, Germany; 12 × 49 mm) was deployed in the infrarenal aorta. Immediately after the procedure, angiography revealed complete occlusion of the left renal artery ostium due to inadvertent stent coverage, and endovascular recanalization was not feasible (Fig. 1C and D). An urgent multidisciplinary consultation was conducted. Considering the short ischemic time (approximately 3 hours), the patient's young age, and the high risk of failure with repeat endovascular intervention in the setting of active vasculitis, emergency renal autotransplantation was selected as the only viable salvage option. The left kidney was harvested through a flank incision using a technique similar to living-donor nephrectomy. Immediate cold perfusion with Custodiol solution was initiated. A short segment of the renal artery containing the occluded stent was resected, and the vessel was found to have preserved intimal integrity. Retrograde venous perfusion was performed to optimize endothelial protection. The graft was then transplanted into the right iliac fossa, with end-to-side anastomoses to the external iliac vessels. Ureteroneocystostomy was performed using the Lich–Gregoir technique with ureteral stent placement. Total operative time was 190 minutes with an estimated blood loss of 100 mL. Warm ischemia time was approximately 3 hours, and cold ischemia time was 45 minutes (Fig. 2). Postoperatively, the patient was admitted to the intensive care unit in stable condition, requiring brief vasopressor support. Urine output reached 4000 mL within the first 24 hours, although serum creatinine transiently increased to 187 μmol/L. A perigraft hematoma was detected on postoperative day 1 and was successfully managed with a single ultrasound-guided percutaneous aspiration without impairing graft perfusion. Renal function progressively improved, with serum creatinine decreasing to 92.6 μmol/L by postoperative day 10 and 74 μmol/L by day 30. Follow-up Doppler ultrasound demonstrated good graft perfusion, and the ureteral stent was removed uneventfully. At 60 days, renal function remained stable (serum creatinine 95 μmol/L) with preserved graft perfusion parameters (Fig. 3). No major complications such as thrombosis, infection, or graft dysfunction were observed during follow-up.

**Fig. 1:**
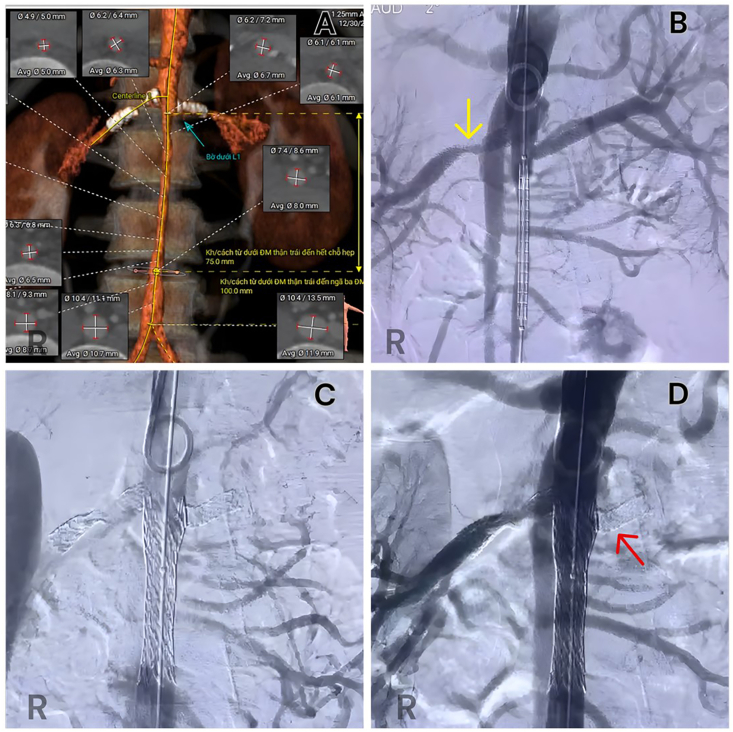
**Computed tomography angiography and digital subtraction angiography images**. (A) Pre-intervention three-dimensional computed tomography angiography demonstrating bilateral renal artery stents and infrarenal aortic stenosis. (B) Intraoperative Digital Subtraction Angiography. The yellow arrow illustrates right renal artery stenosis, but blood supply remains adequate. (C) Post-intervention digital subtraction angiography following deployment of the covered aortic stent. (D) Delayed angiographic image demonstrating persistent occlusion of the left renal artery ostium (red arrow).

**Fig. 2:**
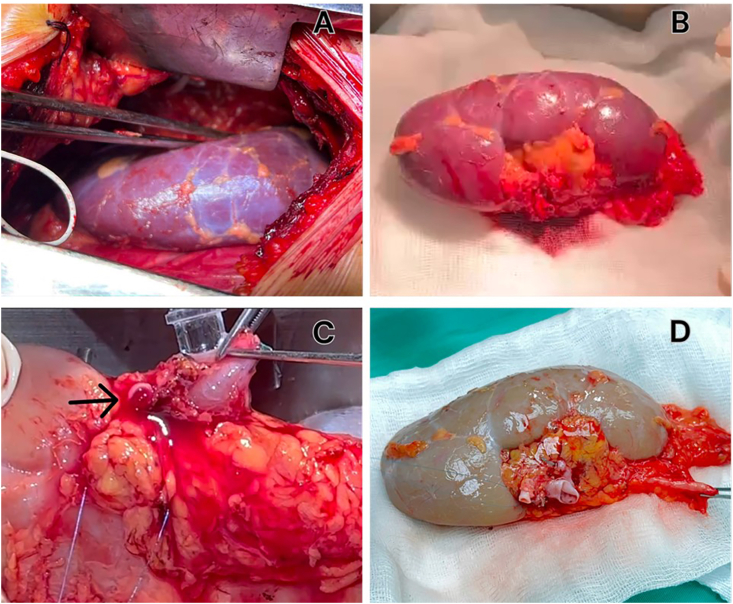
**Retrograde perfusion of the renal vein and artery with cold Custodiol solution to remove microthrombi and preserve arterial endothelial integrity**. (A) Left kidney ischemia at 4th hour. (B) Kidney after removed from the renal fossa. (C) Retrograde perfusion by Custodiol solution. The black arrow indicates renal artery. (D) Kidney after cold perfusion.

**Fig. 3:**
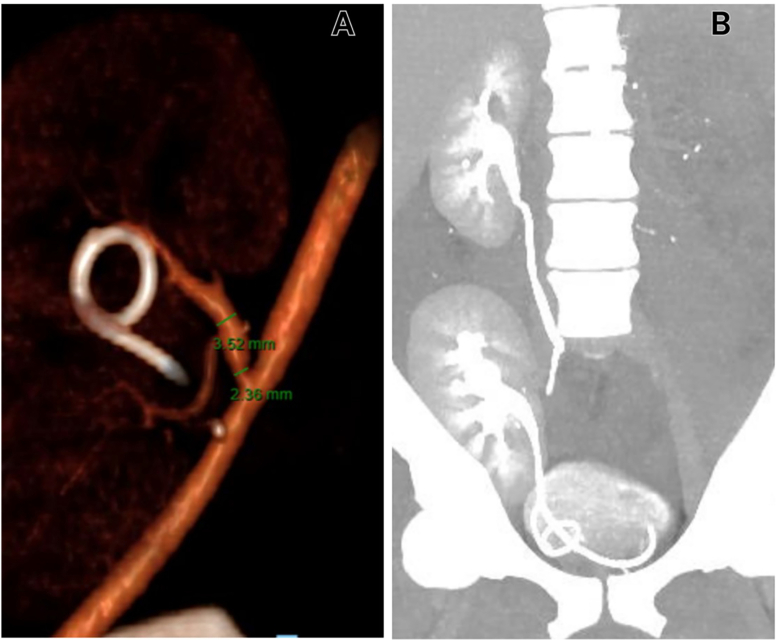
**Postoperative imaging and recovery of renal function** (A) Three-dimensional computed tomography angiography demonstrating a patent renal arterial anastomosis and preserved graft perfusion. (B) Computed tomography urography demonstrating unobstructed urinary drainage and preserved function of the autotransplanted kidney.

## 3 Discussion

This case demonstrates that emergency renal autotransplantation (RAT) can serve as an effective salvage strategy in acute renal artery occlusion following iatrogenic aortic intervention. In contrast to more common complications of endovascular treatment in Takayasu arteritis (TAK), this case involved complete mechanical occlusion of the renal artery ostium caused by inadvertent aortic stent coverage, resulting in immediate cessation of renal perfusion. Under these circumstances, endovascular rescue was not feasible, as the stent itself prevented catheter access to the renal artery. Although *in situ*renal artery bypass may be considered in some cases of renal vascular stenosis, it was not favored in the present case. The renal ostium was completely covered by a deployed aortic stent, making reconstruction technically difficult. Additionally, the surrounding aortic segment was affected by active TAK which raised concerns regarding the durability of a bypass graft. Given the ongoing warm ischemia and the need for rapid renal salvage, emergency RAT allowed immediate cold perfusion and urgent revascularization, ensuring the best possible preservation of renal function. Consequently, RAT was the preferred strategy under the specific conditions of this case. A critical factor contributing to the successful outcome was the short ischemic interval. Revascularization was achieved within approximately 3 hours of warm ischemia, which is generally considered within the acceptable window for renal salvage. Immediate ex vivo cold perfusion with Custodiol solution enabled rapid metabolic suppression and conversion to protected cold ischemia, thereby preserving graft viability until reimplantation. The early recovery of urine output and normalization of renal function further support the effectiveness of this time-sensitive strategy.

RAT has traditionally been described in elective settings, particularly for complex renovascular disease or chronic renal artery stenosis ^5^ In contrast, its application as an emergency salvage procedure following acute iatrogenic vascular injury remains rarely reported. This distinction highlights a broader and underrecognized role for RAT beyond planned reconstruction. Previous experience at our institution has also demonstrated the utility of RAT in other complex reconstructive scenarios, such as complete ureteral loss ^6^ Reported success rates of 85–90% when warm ischemia is maintained under 4 hours further support its role in carefully selected cases ^5^

The postoperative course in this patient further confirms the technical success of the procedure. Renal function recovered progressively, and follow-up imaging demonstrated preserved graft perfusion without evidence of early vascular compromise. Although peak systolic velocity in the graft artery was elevated, the resistive index remained within normal limits, suggesting the absence of clinically significant dysfunction. These findings indicate that favorable short-term outcomes can be achieved even in high-risk vascular settings ^7^

This case also underscores several important considerations in the management of TAK. First, TAK should be considered in young patients presenting with unexplained hypertension or vascular abnormalities. Delayed diagnosis is not uncommon, particularly in male patients, in whom the disease is less frequently recognized ^8^^,^^9^ Second, assessment of disease activity remains challenging, as conventional inflammatory markers such as CRP and ESR may not reliably reflect disease progression ^9^^,^^10^ In the present case, preoperative imaging findings suggestive of active vasculitis likely contributed to the increased risk of procedural complications, including stent malposition and acute thrombosis.

Finally, the systemic nature of TAK necessitates ongoing medical management even after successful surgical intervention. While the left kidney was salvaged, the presence of in-stent stenosis in the contralateral renal artery remains a concern and requires close surveillance. Long-term outcomes depend on strict adherence to immunosuppressive and antithrombotic therapy, as well as regular imaging follow-up.

In summary, this case highlights a rare but severe iatrogenic complication of endovascular aortic intervention and demonstrates that emergency RAT can be a life-saving option for renal preservation. Early recognition, rapid decision-making, and a multidisciplinary approach are essential to optimize outcomes when conventional endovascular rescue is not technically feasible.

## 4 Conclusion

This case highlights that emergency renal autotransplantation is a viable salvage option for acute renal artery occlusion following aortic stent coverage in Takayasu arteritis. In situations where endovascular recanalization is not feasible, prompt recognition and timely multidisciplinary intervention are critical to preserve renal function.

## CRediT authorship contribution statement

**Hung Ngoc Pham:** Writing – review & editing, Supervision, Project administration, Conceptualization. **Anh Van Quoc Nguyen:** Investigation, Data curation. **Can Van Truong:** Investigation. **Tuan Kim Nguyen:** Visualization, Investigation. **Viet Huu Quoc Phan:** Data curation. **Tuan Minh Truong:** Investigation. **Phuc Dai Hong Vo:** Resources. **Hieu Van Le:** Investigation. **Xuan Thi My Hoang:** Visualization. **Dung Duc Nguyen:** Writing – review & editing, Validation, Supervision. **Kha Cong Le Bui:** Writing – review & editing, Writing – original draft, Formal analysis, Conceptualization.

## Funding

This research received no external funding.

## Declaration of competing interest

The authors declare that they have no known competing financial interests or personal relationships that could have appeared to influence the work reported in this paper.
